# Computational approach towards identification of pathogenic missense mutations in *AMELX *gene and their possible association with amelogenesis imperfecta

**DOI:** 10.22099/mbrc.2020.35413.1456

**Published:** 2020-06

**Authors:** Narendra Shivani, Aseervatham Selvi Smiline-Girija, Arumugam Paramasivam, Jayaseelan Vijayashree-Priyadharsini

**Affiliations:** 1 Saveetha Dental College, Saveetha Institute of Medical and Technical Sciences (SIMATS), Saveetha University, Chennai, India; 2 Department of Microbiology, Saveetha Dental College, Saveetha Institute of Medical and Technical Sciences (SIMATS), Saveetha University, 162, Poonamallee High Road, Chennai 600077, Tamil Nadu, India; 3 Biomedical Research Unit and Laboratory Animal Centre-Dental Research Cell, Saveetha Dental College, Saveetha Institute of Medical and Technical Sciences (SIMATS), Saveetha University, Chennai 600077, India

**Keywords:** Mutation, AMELX gene, Enamel, Amelogenesis imperfecta, In silico

## Abstract

Amelogenin gene *(AMEL-X)* encodes an enamel protein called amelogenin, which plays a vital role in tooth development. Any mutations in this gene or the associated pathway lead to developmental abnormalities of the tooth. The present study aims to analyze functional missense mutations in *AMEL-X *genes and derive an association with amelogenesis imperfecta. The information on missense mutations of human *AMEL-X *gene was collected from Ensembl database (https://asia.ensembl.org). Three different computational tools viz., SIFT, PolyPhen and PROVEAN were used to identify the deleterious or pathogenic forms of mutations in the gene studied. I-Mutant Suit was used to identify the stability of the proteins identified as deleterious by the three tools. Further, MutPred analysis revealed the pathogenicity of these mutations. Among 96 missense variants reported in *AMEL-X* gene, 18 were found to be deleterious using the three prediction tools (SIFT, PolyPhen and PROVEAN). When these variants were subjected to protein stability analysis, about 14 missense variants showed decreased stability whereas the other 8 variants showed increased stability. Further, these variants were analyzed using MutPred which identified 9 variants to be highly pathogenic. ExAC database revealed that all the pathogenic mutations had a minor allele frequency less than 0.01. The *in silico* analysis revealed highly pathogenic mutations in amelogenin gene which could have a putative association with amelogenesis imperfecta. These mutations should be screened in patients for early diagnosis of susceptibility to AI.

## INTRODUCTION

Development of tooth is an orchestrated complex process involving multiple molecular and cell-to-cell interactions. Amelogenesis imperfecta (AI) is a heterogenous group of genetic disorders characterized by hypoplasia and/or hypomineralization of the dental enamel in both primary and permanent dentition [1]. The prevalence of AI ranges from 1:700 to 1:14,000 varying across different populations. A study reports a prevalence of 0.02% in southern district of Andhra Pradesh [2]. More than 13 different classification systems based on phenotypes are documented [3]. Nonsyndromic or isolated forms of amelogenesis imperfecta (AI) are reportedly due to 20 different mutations [4]. AI is found to be inherited commonly as an X-linked disorder, although autosomal dominant and recessive patterns have also been suggested. Mutation of *AMELX* encoding amelogenin, present at the cytogenetic loci Xp22.3 – p22.1, has been attributed as a cause of AI [5]. Autosomal dominant and recessive patterns are found to be contributed by defects in the enamelin gene (*ENAM* 4q21) [6]. A locus on 4q13.3 has been associated with recessive form of inheritance [7]. A recent study identified a novel mutation in *RELT *gene that is a member of the tumor necrosis factor receptor superfamily (TNFRSF) to be associated with autosomal recessive form of AI [8].

Enamel matrix proteins govern the process of enamel mineral initiation, elongation and organization. The amelogenin gene of size 9 kilobases consisting of seven exons codes for the amelogenin protein. Several types of mutations such as deletion of a part of the gene, missense, non-sense mutations have been identified in this gene. Specific regions on the genes are responsible for controlling the enamel thickness while other regions play an important role in enamel mineralization [9]. The enamel is most often hypoplastic, hypomineralized with discoloration, sensitivity and prone to disintegration. AI has been reported as an isolated syndrome or as an abnormality linked to syndromes [10]. Aesthetic and functional problems associated with AI demands early detection of this hereditary disorder. Hence the present study was designed to identify potential functional mutations which may be associated with AI employing computational tools.

## MATERIALS AND METHODS

**Data collection:** The rationale behind choosing the AMELX for the present study is that, it is one of the few enamel matrix protein available which can be directly associated with the disease phenotype AI. Hence, it is imperative to identify pathogenic mutations in *AMELX *gene to have a more vivid picture of the pathogenesis of AI. The information on mis-sense mutations of human *AMELX* gene was collected from Ensembl database (https://asia.ensembl.org) [11]. As of January 2019, 96 missense mutations were screened using three different computational tools viz., SIFT, PolyPhen and PROVEAN. The curated data obtained from the three tools were further analyzed using I-Mutant and MutPred to identify the stability of protein variants and their pathogenicity respectively. The description of individual softwares has been discussed below.

**SIFT analysis: **The Sorting Intolerant From Tolerant tool employs multiple sequence alignment information to predict the mutations which are tolerated and deleterious at every position of the query sequence. Substitutions located at each position with normalized probabilities less than tolerance index of 0.05 are considered to be intolerant or deleterious, while those which are greater than 0.05 are considered to be tolerated [12].

**PolyPhen analysis: **PolyPhen-2 (Polymorphism Phenotyping v2), predicts the possible outcome of amino acid substitutions on the stability and function of human proteins using structural and comparative evolutionary considerations. It estimates the probability of the missense mutation being damaging based on a combination of functional annotation of single-nucleotide polymorphisms (SNPs), maps coding SNPs to gene transcripts, extracts protein sequence annotations and structural attributes, and builds conservation profiles [13]. 

**PROVEAN analysis:** PROVEAN (Protein Variation Effect Analyzer) is a software tool which predicts whether an amino acid substitution or indel has an impact on the biological function of a protein. PROVEAN is useful for filtering sequence variants to identify nonsynonymous or indel variants that are predicted to be functionally important [14].

**I-Mutant analysis:** I-Mutant v3.0 is a support vector machine (SVM)-based tool for the automatic prediction of protein stability changes upon single point mutations. The software’s predictions are based on the protein sequence. The predictions were classified into three classes: neutral mutation (−0.5 ≤ DDG ≥ 0.5 kcal/mol), large decrease (<−0.5 kcal/mol), and a large increase (>0.5 kcal/mol). The free energy change (DDG) predicted by I-Mutant 3.0 is based on the difference between unfolding Gibbs free energy change of mutant and native protein (kcal/mol) [15].

**MutPred analysis:** MutPred v2 is a standalone and web application developed to classify amino acid substitutions as pathogenic or benign in human. The wild-type protein sequence in FASTA format is used for the purpose and the substitution sites identified. The probability of the mutation being deleterious is reported [16, 17].

**ExAC data analysis:** The Exome Aggregation Consortium (ExAC) is a group of investigators seeking to aggregate exome sequencing data from a wide variety of large-scale sequencing projects, to make summary data available for the wider scientific community. These sequences were extracted for public release based on consent, consortium permission, exome data quality, and lack of relatedness with other samples. The ExAC genome data was used to compare between the observed variants documented in the present study with that of reported variants deposited in the ExAC repository [18].

## RESULTS

**Figure 1:**
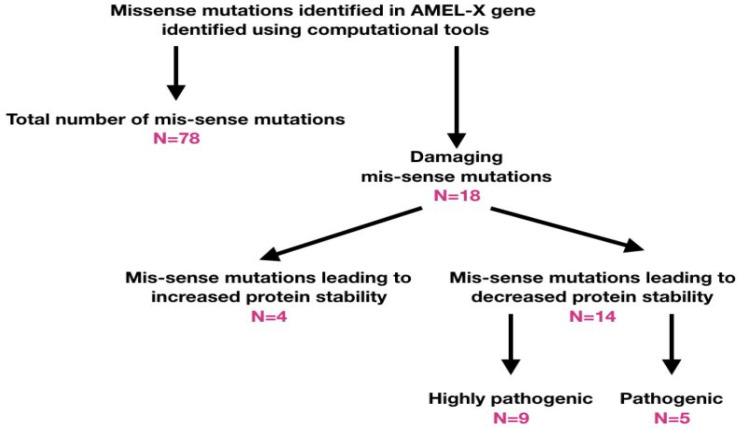
Schematic representation of the present investigation on the missense variants of *AMELX* gene

The list of missense variants in the transcript (ENST00000380712.7) of *AMELX* gene sorted based on their effects as assessed by three prediction tools (SIFT, PolyPhen and PROVEAN) were tabulated. Among 96 missense variants screened, 18 SNPs were found to be damaging as predicted by all the three computational tools described in the methods section (Table 1, Fig. 1). 

The protein structural stability was assessed (Table 2) based on standard free energy change at 25°C with pH 7.0 which was found to increase or decrease based on the protein stability free energy (DDG) change. When DDG>0, there is an increased stability and when DDG<0, there is decreased stability. I-Mutant Suit identified about 14 variants with decreased stability and the other 4 variants showed increased stability. Further, the variants with decreased protein stability were subjected to pathogenicity prediction by MutPred tool. Out of the 14 missense variants 9 were found to be highly pathogenic (MutPred Score > 0.5) and 5 were found to be pathogenic (Table 3). The highly pathogenic variants were assessed for minor allele frequency (MAF). Except for *rs104894738* and *rs104894736* (population data unavailable), all other variants were found to have a MAF<0.01, that clearly indicates that these variants are definite mutations rather than polymorphisms.

**Table 1:** List of missense variants in the transcript (ENST00000380712.7) of *AMELX *gene sorted based on their effects as assessed by three prediction tools (SIFT, PolyPhen and PROVEAN)

**Variant ID**	**AA**	**AA Position**	**SIFT class**	**SIFT score**	**PolyPhen Class**	**PolyPhen Score**	**PROVEAN class**	**PROVEAN score**
rs104894738	W/S	4	deleterious	0	probably damaging	0.999	D	-4.5
rs1020946655	G/R	12	deleterious	0	probably damaging	0.997	D	-2.8
rs1347436776	P/Q	20	deleterious	0	possibly damaging	0.554	D	-2.91
rs1347436776	P/L	20	deleterious	0	probably damaging	0.945	D	-4.1
rs1048604111	P/T	26	deleterious	0	probably damaging	0.987	D	-3.1
rs104894733	T/I	51	deleterious	0	probably damaging	0.999	D	-2.7
rs765738846	W/C	55	deleterious	0	probably damaging	0.999	D	-5.3
rs1188448671	Y/C	56	deleterious	0	probably damaging	0.999	D	-3.8
rs374272157	P/L	64	deleterious	0	possibly damaging	0.893	D	-5.387
rs760691566	Y/C	66	deleterious	0	probably damaging	0.999	D	-3.9
rs104894736	P/T	70	deleterious	0	probably damaging	0.977	D	-4.5
rs1446778772	W/R	74	deleterious	0.02	probably damaging	0.999	D	-6.5
rs780365505	L/P	75	deleterious	0	probably damaging	0.994	D	-2.7
rs751135700	H/D	77	deleterious	0.02	probably damaging	0.998	D	-3.5
rs745822682	H/P	129	deleterious	0.02	possibly damaging	0.667	D	-4.058
rs938995045	P/T	143	deleterious	0.03	probably damaging	0.947	D	-3.972
rs768186118	P/H	183	deleterious	0.03	possibly damaging	0.81	D	-6.661
rs1463899384	P/S	194	deleterious	0.02	probably damaging	0.948	D	-3.555

**Table 2:** Protein structural stability assessment based on standard free energy change at 25°C with pH 7.0

**Variant ID**	**AA**	**AA Position**	**Stability**	**DDG (kcal/mol)**
rs104894738	W/S	4	Decrease	-2.25
rs1020946655	G/R	12	Decrease	-1.45
rs1347436776	P/Q	20	Decrease	-1.36
rs1347436776	P/L	20	Decrease	-1.04
rs1048604111	P/T	26	Decrease	-1.21
rs104894733	T/I	51	Decrease	-1.01
rs765738846	W/C	55	Decrease	-1.47
rs1188448671	Y/C	56	Increase	0.47*
rs374272157	P/L	64	Decrease	-0.27
rs760691566	Y/C	66	Increase	0.77*
rs104894736	P/T	70	Decrease	-1.02
rs1446778772	W/R	74	Decrease	-1.45
rs780365505	L/P	75	Decrease	-1.38
rs751135700	H/D	77	Increase	0.07*
rs745822682	H/P	129	Increase	0.37*
rs938995045	P/T	143	Decrease	-0.24
rs768186118	P/H	183	Decrease	-0.91
rs1463899384	P/S	194	Decrease	-1.42

*Variants which leads to increased stability of the protein (DDG > 0 – Increased stability; DDG < 0 – decreased stability)

## DISCUSSION

Amylogenesis imperfecta is a heterogeneous group of hereditary diseases affecting tooth enamel formation [19]. AI can occur as an isolated form or as a phenotype of syndromic conditions, like enamel-renal syndrome, Jalili syndrome etc., Several genes have been attributed to the disease phenotype, of which mutations in genes encoding enamel matrix proteins have gained more interest due to direct association with AI. *AMELX* (amelogenin), *ENAM* (enamelin), *AMBN* (ameloblastin), *MMP20* (enamelysin) and *KLK4* (kallikrein 4) are some of the genes in which mutations have been identified to be associated with AI. Several other novel genes have also been implicated in the pathogenesis of AI, such as family with sequence similarity 83 member H (FAM83H), solute carrier family 24 member 4 (SLC24A4), chromosome 4 open reading frame 26 (C4orf26) and WD repeat-containing protein 72 (WDR72) [20]. 

**Table 3:** Prediction of pathogenicity of proteins with decreased stability as assessed by MutPred tool

**Variant ID**	**AA**	**AA Position**	**MutPred Score**
rs104894738	W/S	4	0.886**
rs1020946655	G/R	12	0.919**
rs1347436776	P/Q	20	0.613**
rs1347436776	P/L	20	0.569**
rs1048604111	P/T	26	0.577**
rs104894733	T/I	51	0.298
rs765738846	W/C	55	0.563**
rs374272157	P/L	64	0.407
rs104894736	P/T	70	0.586**
rs1446778772	W/R	74	0.531**
rs780365505	L/P	75	0.656**
rs938995045	P/T	143	0.312
rs768186118	P/H	183	0.231
rs1463899384	P/S	194	0.278

** Increased pathogenicity as predicted by MutPred [Score > 0.5 – pathogenic]

The amelogenin protein is encoded by non-allelic genes *AMEL-X* (Xp22.3) and *AMEL-Y* (Yp11.2). Majority of the expression of amelogenin is derived from *AMEL-X*, which is found within the intron 1 of *ARHGAP6* gene. Several studies have identified novel mutations in *AMEL-X* gene with different roles to play in producing the disease phenotype. Kim and his colleagues [21] identified a novel missense mutation in exon 6 (c.242C>T) which results in an amino acid change from proline to leucine at position 81 (Pro81Leu). This mutation was found to change the mRNA splicing repertory, as elucidated by minigene splicing assay. A related study demonstrates the fact that alternative splicing of *AMEL-X* gene produces 6 products viz., AML191, which is the most abundant transcript followed by AML175, AML74, AML72, AML58 and AML19. A silent mutation identified in an AI patient (c.120T>C, pAla40Ala) resulted in the inclusion of exon 4 of *AMELX* gene, which is otherwise skipped in the normal mRNA transcript of amelogenin. Functional analysis of this mutation in animal model demonstrated that inclusion of exon 4 induces defects in enamel matrix mineralization [22].

A knock-in mouse model has demonstrated that AML191 is sufficient to achieve the proper thickness of enamel, but the micro-hardness and reduced toughness requires sequestered action of other minor transcripts. Thus appropriate fine tuning of the process requires all the transcripts to act in symphony [23]. A significant finding was reported by Hart et al., 2002, where they studied two mutations in amelogenin gene apart from a missense mutation viz., a frameshift and a stop gained nonsense mutations leading to a premature stop codon which resulted in a hypoplastic phenotype [24]. It was also reported that mutations at the C-terminus of amelogenin tends to alter the mineralization process and that it is crucial for controlling the thickness of the enamel. A large cohort study on 71 families conducted by Wright and his team, 2011, identified mutations in 6 candidate genes to be associated with AI. Among the families tested, 12 families had *FAM83H* mutations (46%), 6 families were identified with *AMELX* (23%) and 3 families with *ENAM* mutations (11%). Experiments on animal models suggest that knockout mice lacking amelogenin have hypoplastic enamel. Enhanced expression induced by transgenes encoding amelogenin splice variants was found to recover only ~80% of molar and ~40% of the incisor thickness [25]. All the evidences and reports discussed above provide a vivid picture on the functionality of amelogenin protein and its role in the process of AI.

A very recent report identified a novel mutation c185delC in exon 5 of *AMELX* gene resulting in a frameshift p.Pro62ArgfsTer47 [26]. The mutation was confirmed to be homozygous in the proband, hemizygous in father and heterozygous in mother by whole exome analysis. This study provided an insight into the skewing of X inactivation process in relation to the phenotypic variations observed in heterozygous carriers of AI. In line with the above reports, the present study has provided preliminary data employing data mining process to identify the potentially pathogenic variants of amelogenin gene. Further population based studies are warranted to arrive at a conclusion about the association of these pathogenic variants with AI.

## Conflict of Interest:

None
